# Editorial: How Do Metabolism, Angiogenesis, and Hypoxia Modulate Resistance?

**DOI:** 10.3389/fonc.2021.671222

**Published:** 2021-04-12

**Authors:** Hiroshi Kondoh, Josep Castellvi, Matilde Esther LLeonart

**Affiliations:** ^1^ Geriatric Unit, Kyoto University Hospital, Kyoto, Japan; ^2^ Pathology Department, Vall d´Hebron Hospital, Barcelona, Spain; ^3^ Biomedical Research in Cancer Stem Cells Group, Vall d´Hebron Research Institute (VHIR), Barcelona, Spain; ^4^ Spanish Biomedical Research Network Centre in Oncology, CIBERONC, Madrid, Spain

**Keywords:** cancer stem cell, autophagy, cancer, cancer resistance, therapy

Metabolic alterations were among the first discovered hallmarks of cancer. They were first described 90 years ago when Otto Warburg realized that cancer cells in culture had a relatively increased metabolic rate (the Warburg hypothesis). It has been proposed that the drastic changes seen in cancer metabolism are in part attributed to mutations in the mtDNA, metabolic reprogramming, or mitochondrial dysfunction. However, novel players in cancer metabolism are emerging. In this regard, the review of Fernández et al. describes how lipidic alterations impact cancer prognosis and response to treatment. For example, it has been described that obesity increases the risk of cancer death, possibly due to the consequences of lipid accumulation throughout a lifetime. Lipid accumulation changes the microenvironment and produces chronic inflammation by increasing several cytokines. While the levels of genetic or epigenetic modifications diverge in different cancer types, all cancer cells adapt to drastic microenvironmental conditions. This adaptation entails metabolic reprogramming to cope with scarce nutrients and oxygen. Lipid metabolism sustains cancer initiation and contributes to cancer progression and therapy resistance. The role of lipids has been underestimated, as they have largely been considered scaffolds of biological membranes. In recent decades, the role of lipids in cancer has emerged in parallel to the characterization of lipids as essential components of cell signaling, redox homeostasis control, and energy sources (i.e., ß-fatty acid oxidation).

Moreover, while *de novo* synthesis of fatty acids and cholesterol is restricted to the liver and adipocytes in normal cells, cancer cells can synthesize such components. This altered lipid metabolism affects key steps involved in the metastatic process, like migration, invasion, and angiogenesis, and can also be associated with prognosis. Moreover, Fernández et al. provide a list of preclinical and clinical studies with bioactive compounds from natural sources to target lipid metabolism and associated risk factors in cancer.

Tumor adaptation to hypoxia is another important aspect that modulates resistance in cancer. Hypoxia is a forced situation where oxygen levels are different from normal physiological conditions. Hypoxia occurs in higher or minor levels in most cancers, if not all. The detection of hypoxic areas by clinical imaging would improve cancer chemotherapeutic treatments and optimal radiotherapy planning. The technique of positron emission tomography (PET) measures cancer metabolism and cellular proliferation, but it can also measure blood flow and oxygen use. PET can identify patients who would be good candidates for molecularly targeted chemotherapies and can be used to monitor response to these personalized therapies. The primary PET radiotracers for this are ^18^F-fluorodeoxyglucose (for observation of abnormal energy metabolism) and ^18^F-fluorothymidine (for evaluation of cell proliferation). In clinical trials, the current candidate of PET tracers for hypoxia is ^18^F-labeled fluoromisonidazole. In this context, Lu et al. aimed to design a novel nitroimidazole derivative to detect hypoxic regions in tumors, which is important to predict resistance to therapy. These authors observed that Al^18^F-NOTA-NI is a novel nitroimidazole tracer. The major advantage of this new tracer is the quick elimination from normal tissue and its retention in hypoxic cancer tissue. Therefore, it has great potential in the planning of new and efficient therapies.

To identify the new molecular markers of hypoxia with translational relevance, Zhang et al. describe the increase in the expression of the non-coding RNA Inc-NEAT1 under hypoxic conditions in the hepatocellular carcinoma model (HCC), which is among the tumors with the worst life expectancies. Using *in vitro* (i.e., RNA immunoprecipitation and luciferase reporter assays) and *in vivo* (mice model) approaches, Zhang et al. characterize the role of the miR-199a-3p/uridine-cytidine kinase 2 (UCK2) axis and its functional association with Inc-NEAT1. These authors propose that the coordinated pathway that involves IncRNA-NEAT1, miR-99a-3p, and UCK2 upregulation in HCC is a potential signaling cascade and contributes to HCC progression under hypoxic conditions, making it a suitable drug target.

On the other hand, cancer stem cells (CSCs) are the most representative cell type resistant to radio and chemotherapies. Espinosa-Sánchez et al. performed an exhaustive review of various canonical and non-canonical CSC pathways. In addition to the pathways associated with the Yamanaka factors, such as Wnt signaling, the Notch pathway, or Sonic Hedgehog; they also added novel signaling players with a relevant role in resistance, which includes the Hippo pathway, NF-KB signaling, and Toll-like receptors. An overview of the current chemotherapeutic drugs against each specific gene comprised in these signaling routes is shown. Furthermore, these authors propose that cross-talk among different CSC pathways drives the resistance to single pathway inhibitors, enabling CSCs to maintain their CSC phenotype. This aspect should be considered in CSC-directed therapies.

To identify possible cancer markers at the preventive level, Feliciano et al. reported a microRNAs signature (miR-125b, miR-29c, miR-16, miR-1260, and miR-451) in the serum of breast cancer patients that can distinguish women with cancer from healthy individuals. Most of the microRNAs included in the genetic signature (predictor) are related to stemness and resistance. This is the case for miR-125b, miR-29c, miR-16, and miR-451, which have been associated with resistance in various cancer types and cellular models. Interestingly, the predictor described by Feliciano et al. was able to detect the risk of 11 healthy women to potentially develop breast cancer in the future. Moreover, to corroborate the expression of microRNAs with protein expression levels in serum (proteomic study), the low expression of miR-16 was correlated with elevated levels of the CD44 protein. Feliciano et al. describe the stem-related marker CD44, so far identified in the serum of triple-negative breast cancer patients (the subtype of breast cancer with high mortality), as a marker present in the other subtypes of breast cancer with less aggressiveness (luminal breast cancer A or B). Detecting resistance/stem markers in serum will help predict cancer in healthy individuals and identify which patient subgroups are at most risk of recurrences.

The success of personalized therapy for cancer patients has attracted much attention. Autophagy has a decisive role in several cellular functions, and its dysregulation is associated with cancer progression, tumor-stroma interactions, CSC maintenance, and resistance to therapy. A growing body of evidence shows that autophagy is a key regulator in the tumor microenvironment and cellular drug response. Two extensive review articles have focused on the function of autophagy in cancer resistance. Alvarez-Meythaler et al. reinforce the role of chemotherapeutic treatment failure as the main cause of tumor resistance. If the tumor cell population is extremely heterogeneous, certain cells would not respond properly to standard chemotherapeutical treatments. Tumor heterogeneity comprises different tumor cell subpopulations and the interactions with stroma and other immune or fibroblast cell types. Alvarez-Meythaler et al. propose a model by which autophagy-directed therapies can be determinant to avoid the propagation of resistant cell variants when applied in the first-line therapy in combination with standard chemotherapeutic treatments.

Chavez-Dominguez et al. describe the dual role of autophagy in cancer as a tumor suppressor or oncogenic mechanism. They attributed the dual autophagy function mainly to the evolution of the tumor (tumor stage). Chavez-Dominguez et al. claim that in early tumorigenesis, autophagy acts as a tumor suppressor mechanism aiming to destroy those molecules or organelles defective in proliferative cells. While in advanced tumors – especially when the metastatic spread has reached stage III or IV– autophagy acts as an oncogenic driver. In this case, the interaction of several pathways induced by hypoxia, metabolism, and tumor microenvironment contribute to burst autophagy.

Another aspect to consider is that the tumor microenvironment modulates tumor evolution and growth and can also actively participate in conditioning the therapeutic response. This is because – depending on the cell types involved and the cyto- and chemokines released into the extracellular medium – a pre-metastatic niche can emerge in the tumor microenvironment. In this sense, Benavente et al. propose novel strategies to enhance the antitumor immune response, with a special focus on radiotherapy. Strategies to enhance tumor perfusion can increase tumor immunogenicity. Angiogenesis, desmoplasia, and inflammation promote leakage and compression of tumor vessels. Vascular normalization strengthens the vessel wall by reducing intercellular gaps and improving perfusion. Decompression of blood vessels by depleting cancer-associated fibroblasts or extracellular matrix reperfuses the vessel and increments perfusion. Overall, reprogramming the tumor microenvironment to an immunomodulatory state augments antitumor immunity. This can be especially relevant to optimize treatment immunogenicity, improving patient outcomes.

Lastly, there have been major advances in massive sequencing technology, known as next-generation sequencing (NGS). Fernández-Rozadilla et al. describe how, with its ultra-high throughput, speed, and scalability, NGS allows researchers to scrutinize genetic and gene expression information at an unprecedented level. Fernández-Rozadilla et al. highlight the importance of NGS in the revolution of biomedical research. NGS was developed just over a decade ago. Since then, it has been used not only for the diagnosis and prognosis of tumors but also to identify the best chemotherapy treatments.

Overall, although additional basic and clinical research is needed to identify additional regulatory proteins and how they interact to contribute to cancer resistance, in the medium-short term, we expect the FDA to approve new drugs targeting the revised pathways described in this special issue.

## Author Contributions

HK, JC, and ML have written and approved the final version of this article.

## Funding

This work was supported by ISCIII (Instituto de Salud Carlos III) Ref. FIS PI15/01262 (MELL), co-financed by the European Regional Development Fund (ERDF) and AECC Project GEC Ref. GC16173720CARR (MELL).

## Conflict of Interest

The authors declare that the research was conducted in the absence of any commercial or financial relationships that could be construed as a potential conflict of interest.

